# Differentiation of Cerebellum-Type and Parkinson-Type of Multiple System Atrophy by Using Multimodal MRI Parameters

**DOI:** 10.3389/fnagi.2021.687649

**Published:** 2021-08-03

**Authors:** Bin Cui, Weimin Zheng, Shan Ren, Zhigang Chen, Zhiqun Wang

**Affiliations:** ^1^Department of Radiology, Aerospace Center Hospital, Beijing, China; ^2^Department of Neurology, Dongfang Hospital, Beijing University of Chinese Medicine, Beijing, China

**Keywords:** cerebellum-type of multiple system atrophy, Parkinson-type of multiple system atrophy, voxel-based morphology, regional cerebral blood flow, amplitude low frequency fluctuation

## Abstract

Recent studies have demonstrated the structural and functional changes in patients with multiple system atrophy (MSA). However, little is known about the different parameter changes of the most vulnerable regions in different types of MSA. In this study, we collected resting-state structure, perfusion, and patients with functional magnetic resonance imaging (fMRI) data of cerebellum-type of MSA (MSA-c) and Parkinson-type of MSA (MSA-p). First, by simultaneously using voxel-based morphology (VBM), arterial spin labeling (ASL), and amplitude of low-frequency fluctuation (ALFF), we analyzed the whole brain differences of structure, perfusion, and functional activation between patients with MSA-c and MSA-p. Second, we explored the relationships among structure, perfusion, function, and the clinical variables in patients with MSA. Finally, we extracted the MRI parameters of a specific region to separate the two groups and search for a sensitive imaging biomarker. As a result, compared with patients with MSA-p type, patients with MSA-c type showed decreased structure atrophy in several cerebella and vermis subregions, reduced perfusion in bilateral cerebellum_4_5 and vermis_4_5, and an decreased ALFF values in the right lingual gyrus (LG) and fusiform (FFG). Subsequent analyses revealed the close correlations among structure, perfusion, function, and clinical variables in both MSA-c and MSA-p. Finally, the receiver operating characteristic (ROC) analysis showed that the regional cerebral blood flow (rCBF) of bilateral cerebellum_4_5/vermis_4_5 could differentiate the two groups at a relatively high accuracy, yielding the sensitivity of 100%, specificity of 79.2%, and the area under the curve (AUC) value of 0.936. These findings have important implications for understanding the underlying neurobiology of different types of MSA and added the new evidence for the disrupted rCBF, structure, and function of MSA, which may provide the potential biomarker for accurately detecting different types of patients with MSA and new ideas for the treatment of different types of MSA in the future.

## Introduction

Multiple system atrophy (MSA) is a progressive neurodegenerative disorder, pathologically characterized by deposition of alpha synuclein-positive glial cytoplasmic inclusions (GCIs) in several specific regions including the striatum, cerebellum, and olivopontine structures (Brettschneider et al., 2017; Krismer et al., 2019). Clinically, MSA is mainly divided into MSA-p type with poor levodopa-responsive parkinsonian syndrome, and MSA-c type with cerebellar ataxia syndrome. However, the clinical differentiation between patients with MSA-c and MSA-p is challenged by the presence of clinical features common to both forms, resulting in the relatively high rate of misdiagnosis and incorrect treatment at clinical practice. Previous researchers have applied structural magnetic resonance imaging (sMRI) in aiding the differential diagnosis between MSA-c and MSA-p type (Matsusue et al., 2009; Deguchi et al., 2015; Krismer and Wenning, 2017; Chelban et al., 2019; Dash et al., 2019), and found that structural atrophy was mainly concentrated in the putamen, middle cerebellar peduncle (MCP), pons, and cerebellum. Another study found no structural abnormalities in MSA patients with MSA (Chelban et al., 2019), which inspires us to find some more sensitive technique to reach an accurate differential diagnosis of the disease.

Arterial spin labeling (ASL) is a non-invasive technique that can provide quantitative information of regional cerebral blood flow (rCBF) (Zhang, 2016). Ideally, the use of ASL might show a higher discriminative value than the sMRI technique, because the hypo-perfusion injury always occurs earlier than the structural atrophy. The ASL technique takes the inflowing magnetically labeled arterial blood as exogenous an contrast agent, instead of routine contrast-enhanced MRI (Hartkamp et al., 2013). Moreover, ASL has several other advantages, such as higher spatial resolution, faster acquisition, and stability (Haller et al., 2016). So far, only two MSA studies have focused on the ASL application. One of the previous studies revealed the decreased rCBF in several cerebellum subregions in patients with MSA-c type (Zheng et al., 2019). Another ASL study focused on differentiation of the Parkinson's disease (PD) and MSA, although the authors performed some perfusion comparisons between patients, with MSA-c and MSA-p they did not further extract the imaging biomarkers to classify the different types of MSA (Erro et al., 2020).

Recently, increasing attention has focused on exploring MSA-related intrinsic brain activity changes during resting-state functional MRI (rs-fMRI). For the intrinsic brain activity, by using the regional homogeneity (ReHo) approach, the researcher found the motor network dysfunction in patients with MSA (You et al., 2011). Besides the ReHo method, the amplitude of low-frequency fluctuations (ALFF) played an important role, which can directly reflect regional spontaneous brain activity by calculating the square root of the power spectrum of the rs-fMRI signals in a low-frequency range (Zang et al., 2007). In one of the previous studies, we used the ALFF method to examine the whole brain activity changes and observed significantly decreased ALFF values in several cerebellum regions in patients with MSA-c type (Ren et al., 2019). Another study focused on MSA-p type and revealed decreased ALFF values mainly in bilateral basal ganglion, as well as increased ALFF values in right cerebellum and parieto-temporo-occipital cortex (Wang et al., 2017). These results were based on the comparison between MSA subtype (MSA-p or MSA-c) and normal controls. However, there was no study directly comparing the ALFF changes between MSA-c type and MSA-p type.

Based on the previous studies of structure, perfusion, and function, it trigged the interests in the relationship among the three multimode MRI parameters in different subtypes of MSA. In addition, no study investigated the association among these multimode MRI parameters simultaneously in MSA disease. Additionally, the association between the MRI parameters and clinical performances in patients with MSA is not very clear. Thus, to deeply understand the specific patterns of structure, perfusion, and functional activity in different MSA subtypes, we performed the current study by using the multimodal MRI methods. First, we aim to analyze the whole brain differences of structure, rCBF, and ALFF values between MSA-c and MSA-p. Second, we intend to explore the relationships among perfusion-structure-function coupling of MSA-c and MSA-p. Furthermore, we will explore the correlation between the multimodal MRI parameters and the clinical variables. Finally, we intend to extract the MRI parameters of a specific region to separate the two groups and search for an imaging biomarker of the disease.

## Methods

### Participants

Twenty-four patients with MSA-c type and 13 patients with MSA-p type were recruited at the clinic of Dongfang Hospital of Beijing University of Chinese Medicine. There was no statistical difference in age and gender between the two groups. The diagnosis of MSA-c and MSA-p type was according to the established international diagnostic criteria of probable MSA defined by the American Academy of Neurology and American Autonomic Society (Gilman et al., 2008). All subjects were evaluated by complete physical and neuropsychological examinations including Unified Multiple System Atrophy Rating Scale (UMSARS). The clinical examinations were performed on the day before MRI scanning. The inclusion criteria for controls were as follows: (1) there were no neurological or psychiatric disorders including obsessive disorder, anxiety disorder, schizophrenia, depression, epilepsy, and so on; (2) there was a lack of significant cognitive decline (Mini-mental State Examination (MMSE) score > 24); (3) there were no neurological deficiencies including visual or hearing loss; (4) there were no treatment with deep brain stimulation or operation; (5) there was no evidence of movement disorder, vascular brain lesions, brain tumor, and/or marked cortical and/or subcortical atrophy on MRI scan. The exclusion criteria for the subjects were as follows: the subjects of hemorrhage, infarction, tumors, trauma, or severe white matter (WM) hyperintensity were excluded from the study. Clinical and demographic information of the subjects were shown in Table 1.

**Table 1:** Clinical and demographical data.

	**MSA-c (*n* = 24)**	**MSA-p (*n* = 13)**	***p*-Value**
Age, years	57.29 ± 1.20	54.08 ± 1.96	0.147
Gender, male/female	14/10	4/9	0.115
UMSARS-I	16.54 ± 1.17	16.67 ± 1.60	0.847
UMSARS-II	16.17 ± 1.28	20.27 ± 1.93	0.080
UMSARS-TOTAL	32.71 ± 2.27	36.82 ± 3.51	0.272

*MSA-c, cerebellum-type of multiple system atrophy; MSA-p, Parkinson-type of multiple system atrophy; UMSARS, Unified Multiple System Atrophy Rating Scale*.

All subjects gave written informed consent in accordance with the Declaration of Helsinki. The protocol was approved by the Medical Research Ethical Committee of Dongfang Hospital of Beijing University of Chinese Medicine.

### Data Acquisition

MRI data acquisition was performed on a GE 3.0T Discovery 750 scanner. Foam padding and headphones were used to control head motion and scanner noise. High-resolution anatomical images were collected using a 3D brain volume T1-weighted sequence with the following parameters: repetition time (TR)/ echo time (TE)/inversion time (TI)/ flip angle (FA) = 8,150 ms/3.17 ms/450 ms/12°, resolution = 256 × 256 matrix, slices = 188, thickness = 1 mm, voxel size = 1 × 1 × 1 mm^3^. The 3D-ASL data were acquired using the following parameters: TR/TE = 2.0 s/14 ms, post-label delay (PLD) = 2.0 s, field of view (FOV) = 256 × 256 mm^2^, matrix size = 64 × 64, in-plane resolution = 3 × 3 mm^2^, bandwidth = 2,232 Hz/px, phase partial Fourier = 6/8, echo-planar imaging (EPI) factor = 64. Twelve slices of 6 mm thickness were acquired. The resting-state BOLD image parameters were as following: TR/ TE/ FA = 2,000 ms/30 ms/90°, FOV = 24 × 24 cm^2^, resolution = 64 × 64 matrix, slices = 36, thickness = 3 mm, gap = 1 mm, voxel size = 3.75 × 3.75 × 3 mm^3^, and bandwidth = 2,232 Hz/pixel. All subjects were instructed to keep their eyes closed, relax, move as little as possible, think of nothing in particular, and stay awake during the scans.

### Image Analysis

#### Voxel-Based Morphology (VBM) Analysis

T1-weighted images were preprocessed with the Statistical Parametric Mapping 12 (SPM12) (http://www.fil.ion.ucl.ac.uk/spm/) software implemented in MATLAB 2013a (Math Works, Natick, MA, USA). First, the T1 structural images were segmented into gray matter (GM), WM, and cerebrospinal fluid (CSF) areas, using the unified standard segmentation option in SPM12. Then, images were spatially normalized into the standard Montreal Neurological Institute (MNI) space by employing the Diffeomorphic Anatomical Registration through Exponentiated Lie algebra (DARTEL) algorithm. The normalized GM and WM components were modulated for non-linear change and then smoothed using a Gaussian kernel of 8 mm full width at half-maximum (FWHM).

#### rCBF Analysis

The images with differences in 3D-ASL were averaged, and the rCBF map was calculated with the weighted reference image of proton density (Xu et al., 2010). Image processing was also performed using SPM12 software. First, the rCBF images were normalized to the MNI space. Then, to reduce the individual variance, the normalized rCBF images were standardized using the mean division. Finally, each standardized rCBF map was spatially smoothed with a Gaussian kernel of 8 mm FWHM.

#### ALFF Analysis

Resting-state functional MRI data preprocessing was performed using the Data Processing Assistant for Resting-State fMRI (DPARSF) (Yan and Zang, 2010). The preprocessing steps included data conversion, removal of the first 10 volumes, slice timing, and head motion correction. Head motion between volumes was evaluated and corrected using rigid-body registration, and the dataset with maximum translation exceeding 2.5 mm and maximum rotation exceeding 2.5° were excluded from this study. To spatially normalize the fMRI data, the realigned volumes were spatially standardized into the MNI space using the EPI template. The functional images were resampled into a voxel size of 3 × 3 × 3 mm^3^. Then, the functional images were smoothed with a Gaussian kernel of 4 mm FWHM. Finally, several nuisance covariates (24 motion parameters, their first-time derivations, WM, and CSF) were regressed out from the data. After preprocessing, the effects of low-frequency drift and high-frequency physiological noise (respiratory and cardiac rhythms) were reduced by time bandpass filtering (0.01–0.08 Hz) of the fMRI data. Next, to acquire the power spectrum, the time series was transformed to a frequency domain using fast Fourier transform (FFT) (parameters: taper percent = 0, FFT length = shortest). And then the power spectrum was square-rooted and averaged across 0.01–0.08 Hz at each voxel. This averaged square root was viewed as the ALFF. For standardization purposes, the ALFF value of each voxel was divided by the global mean ALFF value to standardize data across subjects.

### Statistical Analysis

To assess the between-group differences (between the patients with MSA-c and MSA-p) of the rCBF, GM, and WM volumes, and ALFF values in the whole brain, two-sample *t*-tests were performed with age, and gender as covariates using SPM12. The significance threshold was set to Family-wise error (FWE) correction at the cluster level (*p* < 0.05). In addition, multiple comparisons analysis were performed to identify the relationships among perfusion-structure-function coupling of patients with MSA-c and MSA-p type. A note needs to be made here that the multiple comparisons analyze were performed among the average values on regions of interest (ROIs), and the ROIs were obtained by the two-sample *t*-tests of the rCBF, GM, and WM volumes, and ALFF in the whole brain between MSA-c and MSA-p.

Then, a partial correlation analysis was performed with age, and gender as nuisance covariates, to evaluate the associations of the clinical variables with the rCBF, GM, and WM volumes, and ALFF values in the patients with MSA-c and MSA-p type.

Finally, we use receiver operating characteristic (ROC) analysis with SPSS20.0 to obtain a sensitivity and specificity imaging biomarker for the differential diagnosis of patients with MSA-c and MSA-p type.

## Results

### Demographic and Clinical Characteristics

Twenty-four patients with MSA-c and 13 patients with MSA-p type completed the sMRI, ASL, and fMRI scans. Demographic and clinical data for all the participants were shown in Table 1. No statistically significant differences were found between patients with MSA-c and MSAp type in age and gender (*p* > 0.05).

### Differences in Structure, rCBF, and Function Between the Patients With MSA-c and MSA-p

**Figure 1:**
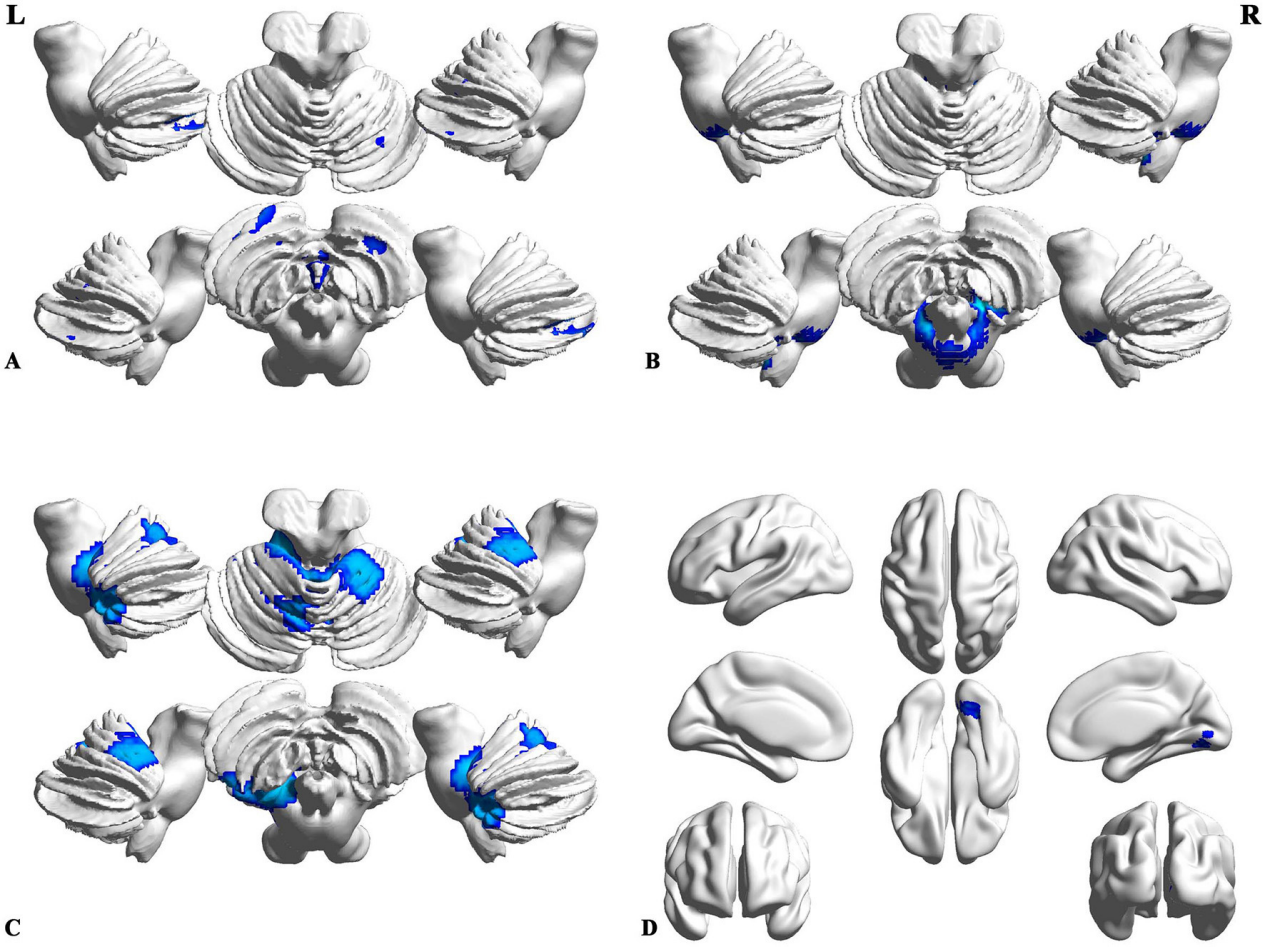
Differences of structure, regional cerebral blood flow (rCBF), and function between the patients with MSA-c and MSA-p. **(A)** Compared to the patients with Parkinson-type of multiple system atrophy (MSA-p type), patients with data of cerebellum-type of MSA (MSA-c type) showed significantly decreased gray matter (GM) volumes in right cerebellum_1, cerebellum_6 and cerebellum_8, left cerebellum_2, cerebellum_1, cerebellum_7b, cerebellum_4_5, vermis_4_5, and vermis_3; **(B)** MSA-c presented significantly decreased white matter (WM) volumes in bilateral cerebellum_9 and left cerebellum; **(C)** MSA-c presented decreased rCBF in the bilateral cerebellum_4_5 and vermis_4_5. **(D)** Decreased amplitude of low-frequency fluctuations (ALFF) values were found in the right lingual gyrus (LG) and fusiform (FFG).

Compared to the patients with MSA-p type, the patients with MSA-c type showed significantly decreased GM volumes (right cerebellum_1, cerebellum_6 and cerebellum_8, left cerebellum_2, cerebellum_1 and cerebellum_7b, left cerebellum_4_5, vermis_4_5, and vermis_3), and WM volumes (bilateral cerebellum_9 and left cerebellum). When compared with patients with MSA-p type, the patients with MSA-c type presented decreased rCBF in bilateral cerebellum_4_5 and vermis_4_5. In addition, we also found the decreased ALFF values in the right lingual gyrus (LG) and fusiform (FFG) in the patients relative to patients. The peak voxels within those significantly different clusters were shown in Figure 1 and Table 2.

**Table 2:** Structural, rCBF, and functional changes between the MSA-c and MSA-p patients.

**Values**	**Brain regions**	**Cluster voxels**	**MNI coordinates (mm)**			**Maximum Z**
			**x**	**y**	**z**	
GMV	Cbe1.R	1,217	24	−69	−47	−4.4975
	Cbe6.R	1,111	32	−73	−42	−4.4975
	Cbe8.R	274	29	−64	−23	−4.4975
	Cbe2.L	930	−32	−75	−39	−5.1103
	Cbe1.L	678	−24	−84	−44	−5.1103
	Cbe7b.L	254	−5	−58	−38	−5.1103
	Cbe4-5.L	381	12	−39	−27	−4.1014
	Ver4-5	148	−2	−52	−18	−4.1014
	Ver3	72	−12	−39	−26	−4.1014
WMV	Cbe9.R	202	−17	−46	−38	−4.3005
	Cbe9.L	106	14	−45	−45	−4.3005
	Cbe8.L	20	2	−21	−41	−4.3005
rCBF	Cbe4-5.L	439	−14	−40	−44	−6.0453
	Cbe4-5.R	406	14	−48	−20	−6.0453
	Ver4-5	240	−14	−32	−24	−6.0453
ALFF	LG.R	37	15	−81	−6	−4.5547
	FFG.R	4	21	−72	−12	−4.5547

*rCBF, regional cerebral blood flow; MSA-c, cerebellum-type of multiple system atrophy; MSA-p, Parkinson-type of multiple system atrophy; GMV, gray matter volume; WMV, white matter volume; ALFF, amplitude of low-frequency fluctuations; Cbe, cerebellum; Ver, vermis; LG, lingual gyrus; FFG, fusiform*.

### Coupling Relationships Among Perfusion-Structure-Function in Patients With MSA-c and MSA-p Type

**Figure 2:**
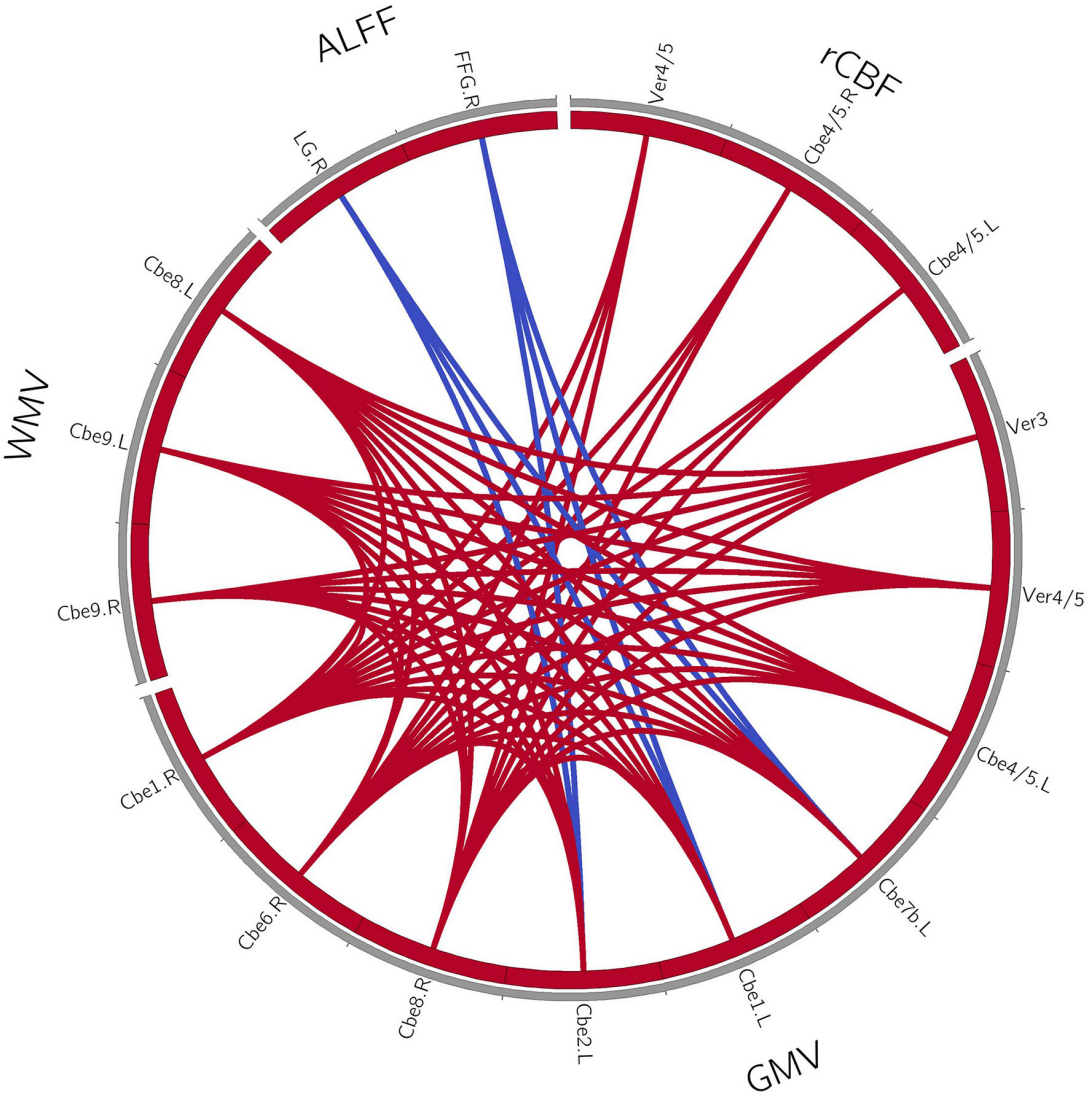
Coupling relationships among perfusion, structure, and function in patients with MSA-c type. For patients with MSA-c type, we found positive correlations within the structure changes, and positive correlations between the decreased rCBF and decreased GM volumes. Furthermore, negative correlations between the ALFF and GM volumes were found. The red line denotes the increased correlations, and the blue line denotes the decreased correlations. GMV, gray matter volume; WMV, white matter volume; rCBF, regional cerebral blood flow; ALFF, amplitude of low-frequency fluctuations; L, left; R, right; Cbe, cerebellum; Ver, vermis; LG, lingual gyrus; FFG, fusiform.

**Figure 3:**
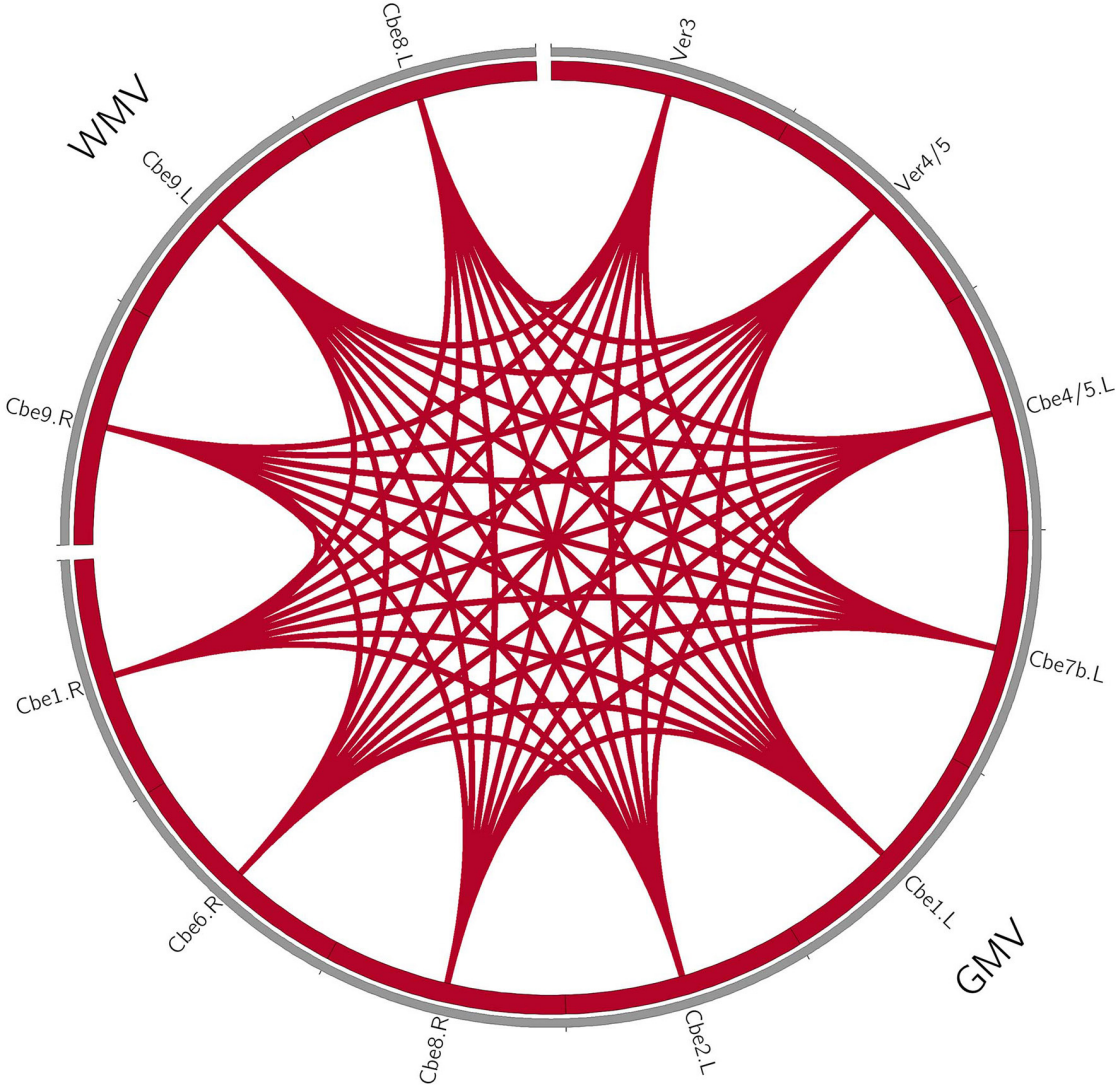
Coupling relationships among perfusion, structure, and function in patients with MSA-p type. For patients with MSA-p type, we only found positive correlations within the structure changes. The red line denotes the increased correlations. GMV, gray matter volume; WMV, white matter volume; L, left; R, right; Cbe, cerebellum; Ver, vermis.

For the patients with MSA-c type, we found the positive correlations within the structure changes (cerebellum and vermis subregions). Furthermore, there were also positive correlations between the decreased rCBF (bilateral cerebellum_4_5, vermis_4_5) and decreased GM volumes (right cerebellum_1, cerebellum_6, cerebellum_8) in patients with MSA-c type. There were negative correlations between the ALFF (right LG/FFG) and GM volumes (left cerebellum_2, cerebellum_1, cerebellum_7b) in patients with MSA-c type (*P* < 0.05, Figure 2). For patients with MSA-p type, we found positive correlations within the structure changes (cerebellum and vermis subregions) (*P* < 0.05, Figure 3).

### Relationships Between Clinical Variables and Multimode MRI Parameters in Patients With MSA-c and MSA-p Type, Respectively

**Figure 4:**
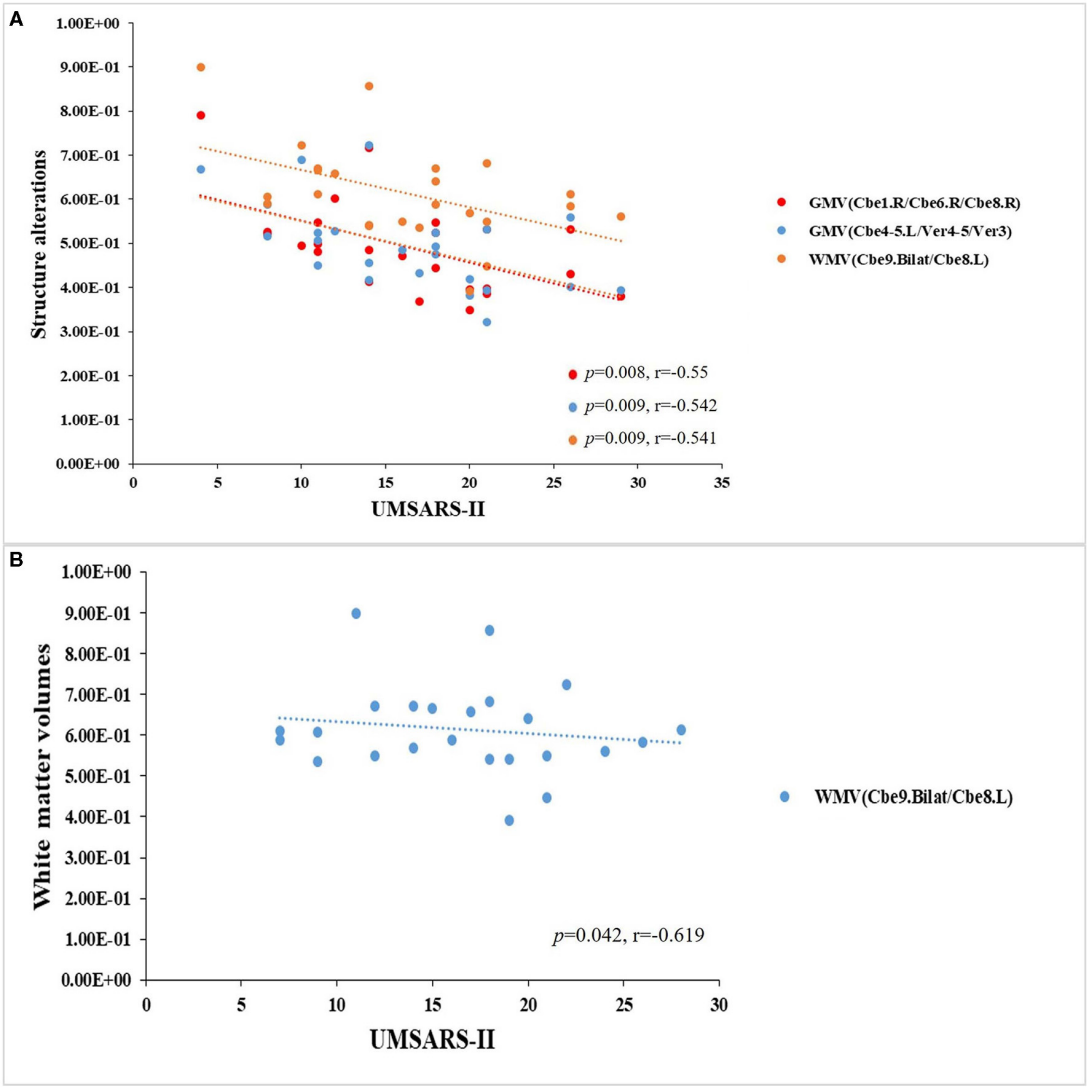
Relationships between clinical variables and multimode MRI parameters in patients with MSAc and MSA-p type, respectively. **(A)** In the MSA-c type group, we found significant negative correlations between the UMSARS -II scores and structure changes. **(B)** In the MSA-p type group, we found a negative correlation between the UMSARS -I scores and WM changes.

In the MSA-c type group, we have found significant negative correlations between the UMSARS-II scores and structure changes (*P* < 0.05, Figure 4A). In the MSA-p type group, we have found a negative correlation between the UMSARS -I scores and WM changes (*P* < 0.05, Figure 4B).

### ROC Analysis

**Figure 5:**
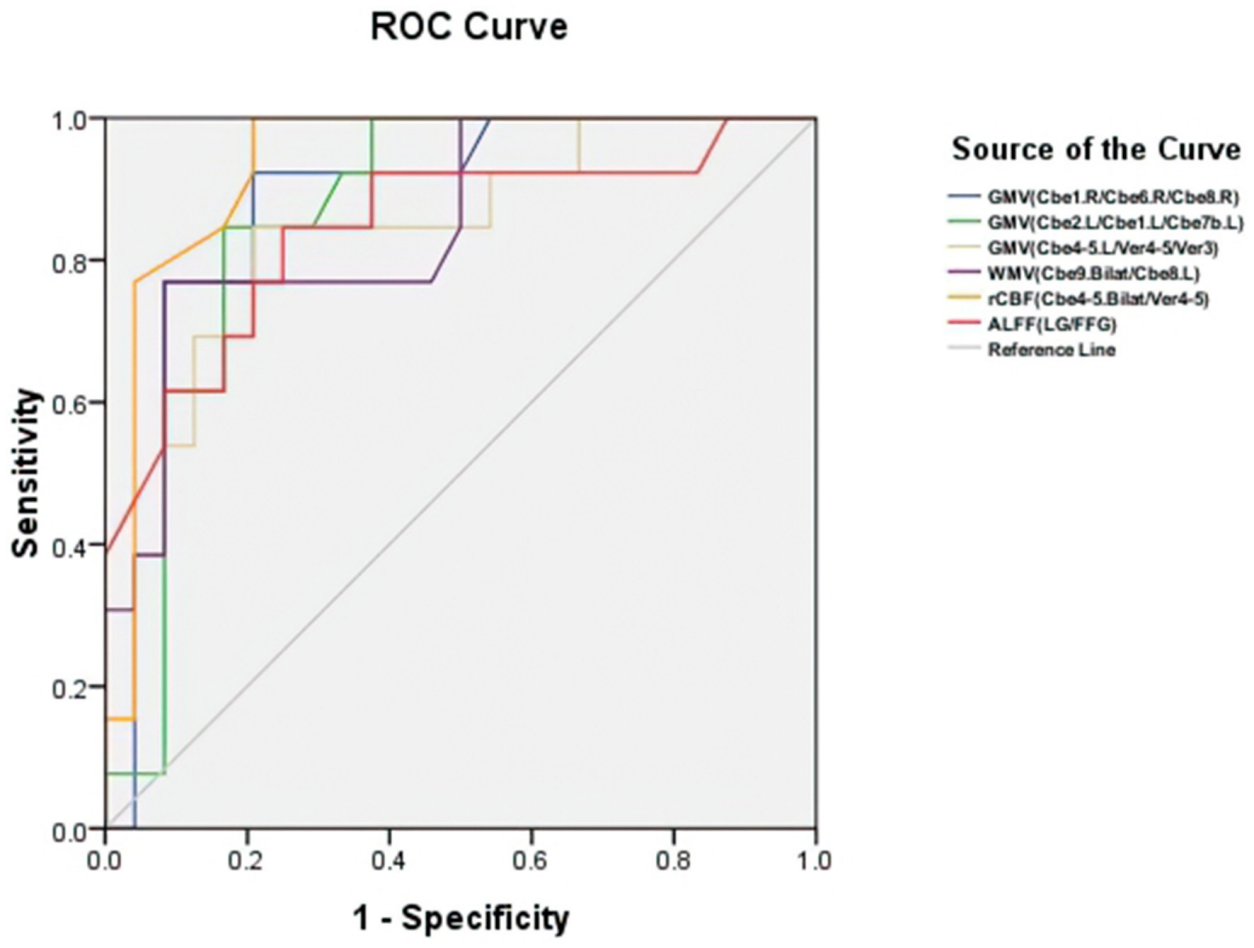
Receiver operating characteristic (ROC) curve for structure, rCBF, and functional alterations in patients with MSA-c and MSA-p. The two groups can be differentiated with a sensitivity of 100% and specificity of 79.2% by using the rCBF values in cerebellum subregions (bilateral cerebellum_4_5) and vermis_4_5, the area under the curve (AUC) of rCBF biomarker was 0.936. By using the GM volume changes in cerebellum and vermis subregions (right cerebellum_1/ cerebellum_6/cerebellum_8, left cerebellum_2/cerebellum_1/cerebellum_7b, and left cerebellum_4_5/vermis_4_5/vermis_3), sensitivities reached 92.3%/84.6%/84.6% and specificities reached 79.2%/83.3%/79.2%, the AUC was 0.880/0.864/0.833, respectively. By using the WM volume changes in bilateral cerebellum_9/ left cerebellum_8, sensitivity reached 76.9% and specificity reached 91.7%, the AUC was 0.851. By using the functional change in right LG/FFG, sensitivity reached 84.6% and specificity reached 75%. The AUC of the function biomarker was 0.845. GMV, gray matter volume; WMV, white matter volume; rCBF, regional cerebral blood flow; ALFF, amplitude of low-frequency fluctuations; L, left; R, right; Bilat, bilateral; Cbe, cerebellum; Ver, vermis; LG, lingual gyrus; FFG, fusiform.

Figure 5 and Table 3 showed the sensitivity and specificity of the rCBF, structural, and functional changes in MSA-c and MSA-p patients. We differentiated the two groups with a sensitivity of 100% and specificity of 79.2% by using the rCBF values in bilateral cerebellum_4_5/vermis_4_5, the area under the curve (AUC) of the rCBF biomarker was 0.936. By using the GM volume changes in cerebellum and vermis subregions (right cerebellum_1/ cerebellum_6/cerebellum_8, left cerebellum_2/cerebellum_1/cerebellum_7b, and left cerebellum_4_5/vermis_4_5/vermis_3), sensitivities reached 92.3%/84.6%/84.6% and specificities reached 79.2%/83.3%/79.2%, the AUC was 0.880/0.864/0.833, respectively. By using the WM volume changes in bilateral cerebellum_9/ left cerebellum_8, sensitivity reached 76.9% and specificity reached 91.7%, the AUC was 0.851. By using the functional change in LG/FFG, sensitivity reached to 0.6% and specificity reached 75%. The AUC of the function biomarker was 0.845.

**Table 3:** Receiver operating characteristic (ROC) curve for structure, rCBF, and functional alterations in MSA-c and MSA-p patients.

**Test result variable(s)**	**AUC**	**Sensitivity (%)**	**Specificity (%)**	***P-*value**	**Cut-off**
GMV (Cbe1.R/Cbe6.R/Cbe8.R)	0.880	92.3	79.2	0.00016	0.715
GMV (Cbe2.L/Cbe1.L/Cbe7b.L)	0.864	84.6	83.3	0.00031	0.679
GMV (Cbe4-5.L/Ver4-5/Ver3)	0.833	84.6	79.2	0.00094	0.638
WMV (Cbe9.Bilat/Cbe8.L)	0.851	76.9	91.7	0.00050	0.686
rCBF (Cbe4-5.Bilat/Ver4-5)	0.936	100	79.2	0.00002	0.792
ALFF (LG/FFG)	0.845	84.6	75.0	0.00100	0.596

*GMV, gray matter volume; WMV, white matter volume; rCBF, regional cerebral blood flow; ALFF, amplitude of low-frequency fluctuations; L, left; R, right; Bilat, bilateral; Cbe, cerebellum; Ver, vermis; LG, lingual gyrus; FFG, fusiform*.

## Discussion

### Major Findings

By applying VBM, ASL, and ALFF analysis to the resting state data acquired from patients with MSA-c and MSA-p, we observed significant structure atrophy in several cerebellum and vermis subregions decreased perfusion in bilateral cerebellum_4_5/vermis_4_5, and decreased ALFF values in the right LG/FFG in patients with MSA-c type, relative to patients with MSA-p type. Subsequent analyses revealed the close correlations among structure, perfusion, and function in patients with MSA-c or MSA-p type. Then, we found significant negative correlations between the UMSARS scores and structure changes in patients with both MSA-c and MSA-p type. Finally, according to the ROC analysis, we found the rCBF value of bilateral cerebellum_4_5/vermis_4_5 could be used as a sensitive biomarker to differentiate MSA-c from patients with MSA-p, with a sensitivity of 100% and specificity of 79.2%. These findings may improve the understanding of the neural pathophysiology mechanisms of different types of MSA from the perspective of perfusion-structure-function coupling.

### Differences in Structure, rCBF, and Function Between the Patients With MSA-c and MSA-p

The cerebellum plays an important role in sensorimotor circuits, which receives information from the spinal cord, trigeminal nerve, vestibule, cerebral cortex, parietal tectum, and sends it to the motor-related regions to regulate movement (Timmann and Daum, 2010). As we knew from the cerebello-cortical circuit, the afferent fibers of the cerebellum form the middle and lower cerebellar peduncles, transferring information mainly from the opposite cerebello-pontine nucleus and the inferior olivary nucleus to the neocerebellum and the efferent fibers of the cerebellum form the main body of the superior cerebellar peduncles, sending information from the neocerebellum to the contralateral thalamus and cerebral cortex. From the view of this process, many cerebella subregions played important roles in the sensorimotor network, which were responsible for the balance, planning, and coordination of motor functions. Early studies believed that the main function of the cerebellum was motor control (Andermann et al., 1975; Takagi et al., 1998). In recent years, many neuroanatomies, neuroimaging, and clinical studies have shown that different subregions of the cerebellum and vermis participate in the different functions, including learning, cognition, emotions, and behaviors (Strick et al., 2009; Gruol et al., 2012; Reeber et al., 2013).

In this study, we observed significant structure atrophy and decreased rCBF in several cerebellum and vermis subregions in patients with MSA-c type relative to patients with MSA-p type. Typical histopathological findings of MSA-c type have been observed predominantly in the cerebellum. Structurally, a recent study revealed the disrupted structural atrophy in the cerebellum of the MSA-c type, including GM loss and WM degeneration (Dash et al., 2019), which was consistent with the current study. Interestingly, we found that the brain areas with reduced perfusion overlapped most of the brain areas with structural atrophy, indicating that decreased perfusion may contribute to the structural impairment in patients with MSA. Specifically, the decreased perfusion of patients with MSA-c type may disrupt the vascular clearance ability, which promotes accumulation of alpha synuclein-positive GCIs and generate neurotoxic matter, finally leading to neurodegeneration of cerebellum (Brettschneider et al., 2017). Therefore, we speculated that the vascular dysfunction of the cerebellum caused by disrupted perfusion might lead to the abnormalities of structure in the patients with MSA-c type. Compared to the patients with MSA-p type, the structure atrophy and reduced perfusion of the cerebellum and vermis subregions were more prominent in patients with MSA-c type, which were consistent with the clinical main symptom of cerebellar ataxia of the MSA-c type disease.

In the present study, we found decreased ALFF in the right LG/FFG in patients with MSA-c type relative to patients with MSA-p. The LG/FFG, as visual association cortex, were responsible for making visual associations, episodic memory consolidation, processing visual imagery, and verbal declarative memory (Bremner et al., 2004; Malhi et al., 2007; Fusar-Poli et al., 2009; Tao et al., 2013; Kukolja et al., 2016). Previous studies have shown that visual and memory participated in regulating sensory and motor function (Dault et al., 2003; Cheng et al., 2004; Ramachandran and Altschuler, 2009; Sayenko et al., 2010; Anguera et al., 2011; Seidler and Carson, 2017; Fisher et al., 2019). In one of the previous studies, we also found functional changes in visual-related regions in patients with MSA-c type relative to healthy controls (Zheng et al., 2019). The direct comparison between the two MSA subtype groups suggested that the visual association cortex may be involved in regulating motor function and present significant ALFF differences in the two groups. The exact mechanism remains unclear.

### Relationships Among Structure, Perfusion, Function, and Clinical Performances

A recent study revealed the coupling relationships between structure and function in the human brain (Baum et al., 2020). Another study assessed baseline associations among perfusion and brain structure using linear regression and found that greater whole-brain perfusion loss was associated with worsening brain structure in aging (Staffaroni et al., 2019). In a study combining ASL and fMRI, researchers found that functional brain hubs were identified primarily in the default-mode, insula, and visual regions, which showed striking spatial correlation with rCBF (Liang et al., 2013). These results revealed a close relationship among structure, rCBF, and brain function, which may be helpful to reveal the basic physiological mechanism of human brain interaction.

In this study, according to the correlation analyses among perfusion-structure-function coupling, we found positive correlations within the structure changes in patients with MSAc and MSA-p type separately. Additionally, we found positive correlations between the decreased rCBF and structure atrophy in patients with MSA-c type, which was consistent with the neurovascular coupling hypothesis (Kuschinsky, 1991; Liang et al., 2013; Venkat et al., 2016), indicating that the rCBF changes may contribute to altered structure in the patients with MSA-c type. Besides, negative correlations between the decreased ALFF and structure atrophy in patients with MSA-c were found, which was consistent with the previous studies (Honey et al., 2010; Dacosta-Aguayo et al., 2014). Several studies explored the structure and function interaction and found that the coupling strength may be intrinsically a reflection of mental states (Wang et al., 2015; Huang and Ding, 2016). In the current study, the negative correlations between structure and function suggest the plastic ability of the functional reorganization to resist the structural damage in patients with MSA.

In this study, we found close associations between clinical performances (UMSARS-I/UMSARS-II scores) and structure atrophy in several cerebellum and vermis subregions, suggesting clinical relevance of structural atrophy in different types of MSA. The altered structural atrophy in these regions might be used as imaging markers for tracking disease progression and classification.

### The Biomarker to Differentiate the Two Groups

In the previous study, several researchers used structure changes as biomarkers to differentiate the MSA-c type and healthy controls. For example, a previous study used the “hot cross bun” sign as a biomarker to diagnose MSA-c type, yielding a high specificity of 97%, but its sensitivity was only 50% (Yekhlef et al., 2000). In the current study, we first employed the VBM, ASL, and ALFF analyses on different subtypes of patients with MSA, and then used different parameters to classify the two groups. Results showed that the rCBF of bilateral cerebellum_4_5/ vermis_4_5 could differentiate the two groups at relatively high accuracy, yielding the sensitivity of 100%, specificity of 79.2%, and the AUC value of 0.936. It could be used as a valuable imaging biomarker for the differentiation between patients of MSA-c and MSA-p type.

## Future Considerations

First, in this study, we mainly focused on the motor function changes of MSA, as measured by UMSARS. Some patients with MSA might present cognitive dysfunction with the disease progresses. In the future, we will collect more subjects to evaluate cognitive performances. Second, in addition to the VBM protocol, the current evaluation of neurodegenerative diseases (NNDs) include the use of diffusion MRI (dMRI) techniques that allow the assessment of more detailed microstructural architecture and parameters in brain tissue (Gatto et al., 2019, 2021). We will add this imaging technique in the future work. Furthermore, it is more clinically meaningful to add the genetic and molecular biomarkers to help diagnosis MSA-c from MSA-p, and it is useful to make a more longitudinal and dynamic context, including adaptive and neuroplasticity mechanisms in the context of MSA and other NDDs (Gatto, 2020), we will consider all relevant studies in the future work. Finally, the sample size is relatively small and the two sample size is imbalanced, in the future, a large sample of multimodal MRI data need to be collected to test the current findings and a longitudinal design including the pre-symptomatic patients (which is of great significance for the progress of MSA research) would be performed to elucidate the progressive changes of the different types of patients with MSA.

## Conclusions

In conclusion, by simultaneously applying VBM, ASL, and ALFF analysis to differentiate MSA-c from patients with MSA-p type, we observed significant structure atrophy and perfusion reduced in several cerebellum and vermis subregions and decreased ALFF in visual-related regions in patients with MSA-c type, compared to patients with MSA-p type. In addition, we found coupling relationships among perfusion, structure and function, as well as significant negative correlations between the UMSARS scores and structure atrophy in patients with MSA. Finally, we found the rCBF value of bilateral cerebellum_4_5/-vermis_4_5 could be used as a sensitive biomarker to differentiate MSA-c from MSA-p. These findings have important implications for understanding the underlying neurobiology of different types of MSA and added the new evidence for the disrupted rCBF, structure and function of MSA, which may provide the potential biomarker for accurately detecting different types of MSA and new ideas for the treatment of different types of MSA in the future.

## Data Availability Statement

The datasets presented in this article are not readily available because this data set involves the privacy of the subjects, so it is not convenient to share the data without the consent of the subjects. Requests to access the datasets should be directed to wangzhiqun@126.com.

## Ethics Statement

The studies involving human participants were reviewed and approved by Medical Research Ethical Committee of Dongfang Hospital of Beijing University of Chinese Medicine. The patients/participants provided their written informed consent to participate in this study.

## Author Contributions

ZC and ZW carried out the research project and conceived the study. SR organized the study. BC and WZ executed the results and wrote the first draft of the manuscript. WZ carried out the statistical analysis and designed the study. BC executed the results. ZW reviewed and critiqued the manuscript. All authors contributed to the article and approved the submitted version.

## Conflict of Interest

The authors declare that the research was conducted in the absence of any commercial or financial relationships that could be construed as a potential conflict of interest.

## Publisher's Note

All claims expressed in this article are solely those of the authors and do not necessarily represent those of their affiliated organizations, or those of the publisher, the editors and the reviewers. Any product that may be evaluated in this article, or claim that may be made by its manufacturer, is not guaranteed or endorsed by the publisher.
